# A Meta-Analysis of MiRNA-497 and Prognosis of Hepatocellular Carcinoma

**DOI:** 10.1155/2024/2211179

**Published:** 2024-03-18

**Authors:** Zhiqiang Xie, Qingzhi Hao, Rongrong Zhu, Ruiping Ma

**Affiliations:** ^1^Department of Epidemiology, The First Affiliated Hospital of Shandong First Medical University & Shandong Provincial Qianfoshan Hospital, No. 16766 Jingshi Road, Jinan 250014, Shandong, China; ^2^Department of Peripheral Vascular, Affiliated Hospital of Shandong University of Traditional Chinese Medicine, Jinan 250011, China; ^3^Shandong Qianfoshan Hospital, Cheeloo College of Medicine, Shandong University, Jinan, China

## Abstract

**Background:**

Recently, microRNA-497 (miR-497) has been reported as a prognostic marker for hepatocellular carcinoma (HCC). However, there is no systematic study summarizing these data. Herein, we elucidated the prognostic role of miR497 in HCC by using meta-analysis.

**Methods:**

We systematically searched Embase, PubMed, Web of Science, and, China National Knowledge Infrastructure for relevant studies. The two researchers conducted data extraction and quality evaluation independently. We used hazard ratios (HRs), odds ratios (ORs), and their 95% confidence interval (95% CI) to evaluate the relationship between miR-497 expression level and HCC prognosis.

**Results:**

A total of 6 studies involving 457 participants were included in this meta-analysis. There was a significant association between the lower level of miR-497 expression and the shorter overall survival (HR = 2.17, 95% CI: 1.67–2.84, *P* < 0.001). Meanwhile, patients with low miR-497 expression were more prone to vascular infiltration (OR = 2.73, 95%: 1.79–4.17, *P* < 0.001). However, the lower expression level of miR-497 had no significant correlation with TNM (tumor-node-metastasis) stage (OR = 1.47, 95% CI: 0.17–12.49, *P*=0.47).

**Conclusions:**

MiR-497 might serve as a prognostic biomarker in HCC, but more clinical studies are needed to confirm this view.

## 1. Introduction

Cancer has long been an important factor affecting morbidity and mortality worldwide [1]. Hepatocellular carcinoma (HCC) is one of the most common cancers, with mortality ranking fourth among all cancers and second only to pancreatic cancer [2]. Although the treatment system for HCC is rapidly developing and several drugs have shown clinical efficacy in phase 3 trials in recent years, the 5-year survival rate of HCC is still very low [3, 4]. The difficulty of early diagnosis of HCC and the lack of effective therapeutic drugs for advanced HCC are two major problems affecting the survival of patients with HCC [5, 6]. Therefore, it is necessary to further reveal the pathogenesis of HCC and identify biomarkers with higher specificity and sensitivity for early diagnosis and prognosis monitoring of HCC.

MicroRNAs (miRNAs) are highly conserved, endogenous nonprotein-encoded small molecules with lengths of 21 to 24 nucleotides, which can bind to the target sequence of the 3′-untranslated region of the target mRNAs, causing degradation or translation inhibition of the target mRNAs at the posttranscriptional level [7]. They may participate in a variety of cellular processes, including proliferation, differentiation, and apoptosis [8]. In recent years, miRNAs have been considered potential biomarkers for cancer prognosis because of their robust expression patterns, stability within cancerous samples, and easy assessment by qRT-PCR [9, 10]. MicroRNA497 is a highly conserved miRNA located on human chromosome 17p13.1 [11]. It can inhibit angiogenesis and metastasis by directly inhibiting vascular endothelial growth factor, thus inhibiting tumor growth, invasion, and metastasis [12]. The main targets of miR-497 include YAP1, WEE1, IGF-1R, IF4E, CCND1, and CCNE1 [13, 14]. Previous studies have shown that miR-497 acts as a tumor suppressor in a variety of tumors, such as pancreatic cancer, breast cancer, nerve cell cancer, and kidney cancer [14, 15].

The downregulation of miR-497 has been reported in HCC [16]. Thus, the role of miR-497 in the prognosis of patients with HCC has attracted much attention. Some studies have shown that the low expression level of miR-497 is significantly correlated with the poor prognosis of HCC [13, 17–21]. However, these studies have their limitations, such as small sample sizes or controversial results. Therefore, to further clarify the relationship between miR-497 and the prognosis of HCC patients, we conducted this meta-analysis.

## 2. Materials and Methods

### 2.1. Search Strategy

We systematically searched the databases of PubMed, Web of Science, Embase, and China National Knowledge Infrastructure (CNKI) for studies published in English, Chinese, or other languages up to October 25, 2021, using the following keywords: (“Hepatocarcinoma” or “hepatocellular carcinoma” or “hepatomas” or “liver carcinoma” or “liver cancer” or “HCC”) and (“mirna-497” or “mir-497”). We also examined the references of retrieved studies to avoid omission.

### 2.2. Inclusion and Exclusion Criteria

The inclusion criteria included (1) studies focusing on patients pathologically confirmed as HCC patients; (2) studies focusing on miR-497 expression in HCC tissues; (3) studies with low and high miR-497 expression subgroups; (4) the relationship between the expression level of mi-497 and the prognosis of HCC patients was evaluated; (5) hazard ratio (HR) with 95% CI or survival curves were provided; and (6) the language of the studies was in English or Chinese. The exclusion criteria were as follows: (1) insufficient data or lack of key information on survival results; (2) review, letter, meeting report, or meta-analysis; and (3) nonhuman experiment or ethical disapproval.

### 2.3. Data Extraction and Quality Assessment

The two authors independently extracted useful information from the selected articles. The following information was extracted: published year, name of the first author, detection method of miR-497, sample size, patient age, patient sex, cut-off value, follow-up time, the number of patients with different TNM stages in low and high miR497 expression level groups, vascular infiltration, alpha-fetoprotein (AFP) level, HBV infection, HR, and its 95% CI for overall survival (OS). If the data is not given directly, the data will be extracted through the survival curve. We contacted the authors of the original studies to obtain missing data or clarify ambiguous information. The two authors independently evaluated the included studies according to the Newcastle Ottawa Scale (NOS). A NOS score ≥6 indicates high quality [22]. Any differences in data extraction and quality evaluation were resolved through consultation.

### 2.4. Statistical Analysis

Statistical analyses were performed using Review Manager 5.3 (Cochrane Collaboration, Oxford, UK) and Stata 16.0 (Stata Corp., College Station, TX, USA). All tests were 2-tailed. HR and its 95% CI, obtained directly or indirectly from the survival curve using Engauge Digitizer 4.1, were combined and considered statistically significant at *P* < 0.05. The heterogeneity among studies was tested by a *Q*-test and *I*^2^ value. If *P* > 0.05 and *I*^2^ < 50%, the fixed-effect model was used to calculate pooled HR; otherwise, the random effect model was used. Sensitivity analysis was performed by omitting each study at a time to assess the consistency and stability of the pooled results. The potential publication bias was evaluated using funnel plots, Begg's test, and Egger's test.

## 3. Results

### 3.1. Features of the Included Studies

**Figure 1:**
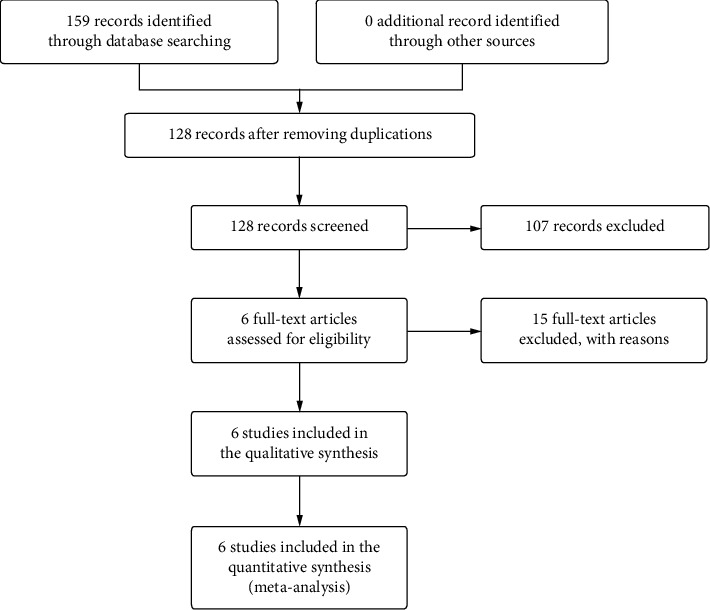
Flow diagram of the search strategy.

As shown in Figure 1, 159 studies were preliminarily searched through the above 4 databases. After removing 31 duplicate articles, the remaining 128 articles were screened according to inclusion and exclusion criteria, and 21 full-text articles were further reviewed. Finally, 6 studies with NOS scores greater than 6 were included after detailed screening [13, 17–21]. HR and 95% CI of two studies were extracted from the survival curve according to the method provided by Jayne F.T et al. The six included studies were all published between 2016 and 2021, including 457 patients. All patients were diagnosed with HCC by histopathology (HE staining) and were of Child-Pugh grade A or B. Homogeneous cancer tissues were resected, and RNA in cancer tissues was extracted and amplified by PCR. With the median level of miR-497 in cancer tissues as the cut-off value, the included patients were divided into the miR-497 high expression group and the miR-497 low expression group.  Table 1 shows the basic characteristics and NOS scores of these studies.

### 3.2. Relationship between the Expression Level of miR-497 and OS

**Figure 2:**
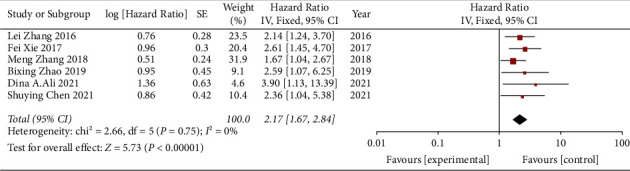
Forest plot of pooled HRs of the high expression level of miR-497 for OS.

As shown in Figure 2, six studies have reported the relationship between miR-497 levels and OS in HCC patients. Since heterogeneity was not obvious (*P*=0.75, *I*^2^ = 0%), we used a fixed-effect model to analyze the low miR-497 expression level and OS. The combined HRs showed that the low expression level of miR-497 was significantly associated with worse OS in patients with HCC (HR = 2.17, 95% CI: 1.67–2.84, *P* < 0.001).

### 3.3. Association between miR-497 Expression Level and Tumor-Node-Metastasis (TNM) Stage

**Figure 3:**
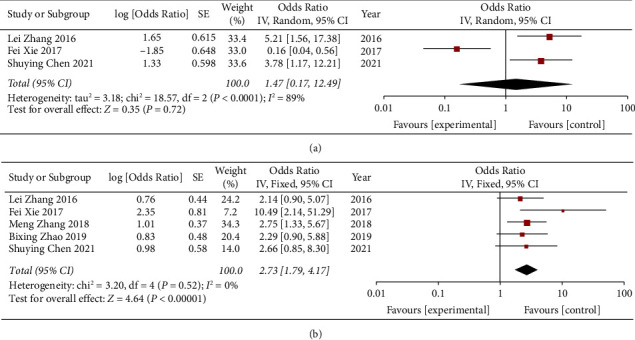
Forest plots evaluating the ORs of miR-497 expression to (a) TNM stage and (b) vascular infiltration.

Three studies reported the TNM classification. Due to the obvious heterogeneity of these reports (*P* < 0.001, *I*^2^ = 89%), we used a random effect model to analyze the combined OR and 95% CI. The analysis showed that the expression of miR-497 had no significant correlation with the tumor-node-metastasis stage (OR = 1.47, 95% CI: 0.17–12.49, *P*=0.72) (Figure 3(a)).

### 3.4. Association between miR-497 Expression Level and Vascular Infiltration

As shown in Figure 3(b), five studies explicitly reported the sample size of vascular infiltration. Because the heterogeneity was not obvious (*P*=0.52, *I*^2^ = 0%), the fixed-effect model was used to analyze the combined OR and its 95% CI. The results showed that patients with low expression of miR-497 may be more prone to vascular infiltration (OR = 2.73, 95% CI: 1.79–4.17, *P* < 0.001).

### 3.5. Relationship between miR-497 Expression Level and Other Clinicopathological Parameters

We also used the combined OR and its 95% CI to analyze the relationship between other pathological indexes of patients with HCC and the expression level of miR-497. In addition to the fixed-effects model for analysis of the age and sex, the random-effects model was selected to analyze all other parameter analyses including AFP level, tumor size, and HBV infection. As shown in Table 2, the combination of OR and 95% CI suggested that the low expression of miR-497 had no significant correlation with pathological parameters such as age, sex, AFP level, HBV infection, and tumor size.

### 3.6. Sensitivity Analysis and Publication Bias

**Figure 4:**
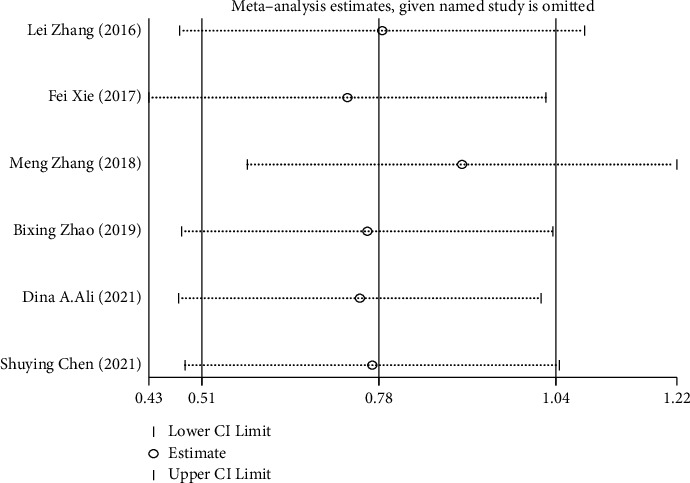
The results of sensitivity analysis.

**Figure 5:**
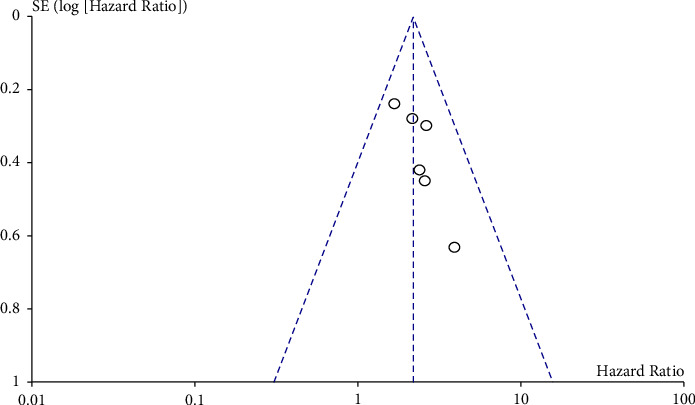
Funnel plot analysis of evaluating publication bias.

To evaluate the credibility and stability of this meta-analysis, we analyzed the sensitivity of the OS group by omitting one study at a time. The results showed that no individual study affected the combined HR for OS (Figure 4). The shape of the funnel plot did not indicate visual evidence of asymmetry (Figure 5). Similarly, Begg's test and Egger's test detected no significant publication bias (*P* > 0.05) (Table 3).

### 3.7. Meta-Regression Analysis

To evaluate the source of heterogeneity in OS, we performed meta-regression. The possible covariates including cut-off, sample size (median 76 as the boundary), NOS (mean 6.83 as the boundary), and ethnicity, were analyzed. The results revealed that these covariates were not the sources of heterogeneity (*P*  >  0.05) (Table 4). This indicates that the pooled results may not be affected by these covariates.

## 4. Discussion

Increasing evidence has confirmed that the disorder of miRNA expression level is involved in the occurrence and progression of various cancers [23], and miRNA expression level is different in normal tissues and tumor tissues [24]. These abnormally expressed miRNAs can play a variety of biological functions in cancer tissues and directly affect the occurrence, development, and metastasis of tumors [25]. Some genes can promote tumorigenesis, such as miR-21 [26], miR155 [27], and miR-222 [28], while others can inhibit tumorigenesis, such as miR-15 [29], miR-139 [30], and mir143 [31]. Therefore, it is a perfect choice to detect the changes of miRNA in tumor tissues by various methods and study its impact on the prognosis of cancer patients.

The occurrence of HCC is a complex process, with unknown etiology, insidious onset, slow early progression, and atypical clinical manifestations. Currently, screening of people at high risk of HCC mainly relies on ultrasonography and AFP detection. However, some small lesions in the early stage are difficult to find by ultrasound, and the AFP level does not increase or has a slight increase in 20%–40% of patients with HCC [32]. Therefore, HCC is often found to be in an advanced stage, which significantly reduces the survival rate of patients with HCC. Liver transplantation, hepatic resection, radiofrequency ablation, transcatheter arterial chemoembolization, targeted therapies based on tyrosine protein kinase inhibitors, and immune checkpoint inhibitors are the most common treatments [2]. However, new treatment approaches for HCC are still needed. In recent years, the relationship between HCC and miRNA expression levels is being studied, expecting to clarify the role of miRNA expression in the diagnosis, treatment, and prognosis of HCC patients.

Mir-497 has always been a research hotspot. Previous studies have shown that miR-497 plays a tumor suppressor role in many tumors. Overexpression of miR-497 can inhibit cell proliferation and induce apoptosis in HCC [17]. However, miR-497 downregulation can promote tumor angiogenesis and contribute to the occurrence of HCC [32]. Although many studies have verified the close relationship between miR-497 and HCC, it is not convincing because of the small sample size. To further clarify the association between miR-497 and HCC, we performed this meta-analysis.

The studies on miR-497 and HCC published after 2016 were searched, and 6 articles, involving 457 patients in total, were included in this meta-analysis. We noted that miR497 expression level significantly affected the OS of HCC patients, and the OS of patients with low expression of miR497 was shorter. When we studied the relationship between miR-497 and a variety of pathological parameters in patients with HCC, we found that the low expression of miR-497 was not significantly correlated with TNM stage, sex, age, AFP level, HBV infection, and tumor size. However, the lower expression level of miR-497 significantly means unfavorable vascular infiltration for HCC patients. Obvious heterogeneity was discovered when we conducted the relationship between miR-497 and a variety of pathological parameters. To evaluate the heterogeneity, we performed a sensitivity analysis. For example, in the sensitivity analysis of the TNM stage, when we omitted the study of Zhang et al. [17], the heterogeneity became negligible. Thus, this article was the main source of heterogeneity. The results of the TNM stage by Xie et al. are not consistent with the combined results of this meta-analysis, which indicates that the relationship between low miR-497 expression level and TNM stage needs further research.

Although this meta-analysis showed that miR-497 level was significantly related to the prognosis of HCC and could be used as a marker to judge the prognosis of HCC patients to a certain extent, it still had some deficiencies and problems. First, only 6 studies were included in this analysis, including only 457 patients, which is not reliable to a certain extent, so more samples are needed to solve it. Second, the dividing value and follow-up time of the original study we included to distinguish the high and low levels of miR-497 are inconsistent, which will lead to large errors in the combination of HR and OR. Therefore, a clear definition and a gold standard are needed to solve this problem. Third, survival data of some eligible studies could not be obtained directly but was extracted from the survival curve, these calculated HRs and their corresponding 95% CIs might also bring several tiny errors. Fourth, this study only proposed that miR-497 was related to the prognosis of liver cancer, but the mechanism and related treatment are still unclear. Fifth, we did not include studies that evaluated the relationship between miR-497 expression and HCC prognosis but did not subdivide miR-497 expression.

## 5. Conclusion

This meta-analysis confirmed that the low expression level of miR-497 was significantly correlated with low OS and more prone to vascular infiltration in patients with HCC. Meanwhile, this study also showed that the expression of miR-497 was not significantly related to the tumor metastasis stage, which was contrary to the views of existing studies and required further research. In conclusion, our findings indicate that the expression level of miR-497 can be used as a marker to predict the OS of HCC patients. More multicenter prospective clinical studies are needed to verify our findings.

## Figures and Tables

**Table 1:** The main characteristics of the studies included in this meta-analysis.

Author	Year	Method	Number	miR-497 expression				Cut-off	Vascular infiltration		Follow-up	Outcome	NOS
				Low		High			Low	High	(months)		
				TNM I∼II	III∼IV	I∼II	III∼IV						
Zhang et al.	2016	qRT-PCR	86	28	15	39	4	Median	24	16	60	OS	7
Xie et al.	2017	qRT-PCR	61	35	5	11	10	Median	21	2	60	OS	7
Zhang et al.	2018	qRT-PCR	125	30	34	41	22	Median	41	25	60	OS	7
Zhao et al.	2019	qRT-PCR	75	—	—	—	—	Median	26	18	30	OS	6
Ali et al.	2021	qRT-PCR	60	—	—	—	—	Median	—	—	—	OS	6
Chen et al.	2021	qRT-PCR	50	9	16	17	8	Median	16	10	60	OS	8

*Note*. The dashes mean no data. qRT-PCR: quantitative real-time PCR; TNM: Tumor-node-metastasis; OS: overall survival; NOS: Newcastle-Ottawa scale.

**Table 2:** The correlation between miR-497 expression and clinicopathologic parameters in HCC.

Clinicopathologic parameters	No. of studies	No. of participants	Pooled OR (95% CI)	*P*	Model	Heterogeneity Chi^2^, *P*, *I*^2^^(%)^
Age	5	397	1.14 (0.76, 1.70)	0.54	Fixed	4.48, 0.35, 11
Sex	5	397	1.12 (0.67, 1.86)	0.66	Fixed	2.93, 0.57, 0
Tumor size (<5 cm/>5 cm)	5	397	0.90 (0.38, 2.11)	0.81	Random	15.7, 0.81, 75
AFP level	5	397	2.11 (0.91, 4.90)	0.08	Random	13.79, 0.008, 71
HBV infection	4	347	0.52 (0.16, 1.69)	0.27	Random	7.13, 0.07, 58

**Table 3:** Publication bias assessed by Begg's test and Egger's test.

Outcome	Begg's test		Egger's test		
	*Z*	*P*	*t*	*P*	95% CI
OS	1.88	0.06	2.78	0.05	0.0268–3.674

OS: overall survival; CI: confidence interval.

**Table 4:** The results of meta-regression analysis of OS.

Covariates	Coefficient	*t*	*P*	95% CI
Cut-off	0.1907	0.40	0.707	−1.119, 1.501
Sample size	−0.3602	−1.32	0.257	−1.117, 0.397
NOS	0.3615	0.92	0.411	−0.732, 1.456
Ethnicity	0.6118	0.95	0.397	−1.179, 2.402
